# Latent profile analysis of sleep quality and its association with psychological resilience among emergency department nurses

**DOI:** 10.3389/fpsyg.2026.1897894

**Published:** 2026-09-28

**Authors:** Ting Wang, Xiaoyu Gou, Yilin Fu, Tongyan Zhang, Xiumin Yin, Ying Peng

**Affiliations:** Shandong Provincial Hospital Affiliated to Shandong First Medical University, Jinan, China

**Keywords:** emergency nursing, latent profile analysis, resilience, sleep disturbance, sleep quality

## Abstract

**Objective:**

Emergency department nurses are consistently exposed to high-intensity and high-pressure working conditions, leading to poor sleep quality. Psychological resilience can help alleviate the negative impacts of work stress. However, few studies have explored how resilience varies across distinct subgroups defined by different sleep profiles. This study employed latent profile analysis to identify distinct sleep-quality profiles among emergency department nurses and explored their association with psychological resilience to provide a basis for targeted interventions.

**Methods:**

From October to December 2025, 611 emergency nurses were recruited from 12 tertiary hospitals in Shandong through convenience sampling. Data were collected using a sociodemographic questionnaire, the Connor–Davidson Resilience Scale, and the Pittsburgh Sleep Quality Index. Sleep quality profiles were determined through latent profile analysis, and associated factors were analyzed using multiple logistic regression.

**Results:**

Emergency department nurses exhibited generally poor sleep quality, which was classified into four distinct profiles: “severely disturbed sleep” (8.0%), “sleep onset difficulty-sleep medication use” (15.1%), “mildly disturbed sleep” (26.7%), and “good sleep quality” (50.2%). Psychological resilience, age, work experience, monthly income, and monthly night shift number were significantly associated with these sleep quality profiles (*p* < 0.05).

**Conclusion:**

Emergency department nurses exhibit distinct sleep quality profiles. Targeted interventions addressing the specific factors associated with each subgroup could improve their sleep outcomes.

## 1 Introduction

Sleep quality is a cornerstone of maintaining physiological homeostasis and psychological well-being, with its importance being particularly pronounced in high-stress and high-demand occupational settings (Sletten et al., 2023; Xia et al., 2026). Adequate sleep supports the normal functioning of the nervous (Xia et al., 2026), immune (You et al., 2024), cardiovascular (Morales-Ghinaglia and Fernandez-Mendoza, 2023) and metabolic (Gonçalves et al., 2022) systems and plays a critical role in emotional regulation (You et al., 2025), cognitive performance (You et al., 2023), and psychological adaptation (de Sá Couto-Pereira et al., 2024). Rhythmic secretion of key hormones such as melatonin and cortisol is closely regulated by the sleep–wake cycle and directly influences alertness, attention, memory consolidation, and stress responsiveness (Souman et al., 2018; Rehman et al., 2019; Patke et al., 2020; Cho et al., 2021). Disruptions in sleep patterns can lead to dysregulation of circadian clock genes (e.g., CLOCK), resulting in desynchronized circadian rhythms, hormonal imbalances, heightened inflammatory responses, and impaired cognitive function—all of which significantly increase the risk of cardiovascular disease, metabolic syndrome, and mood disorders (Xia et al., 2026; Zhu et al., 2026). Thus, addressing sleep-related issues is vital for preserving long-term well-being and optimal brain function.

Emergency department (ED) nurses in clinical practice face exceptional challenges related to sleep due to the unique demands of their work environment and frequent shift rotations, especially night shifts. The fast-paced, unpredictable nature of emergency care, combined with heavy workloads and chronic circadian disruption, places ED nurses at particularly high risk for sleep disturbance (García-Tudela et al., 2023; Bekircan et al., 2025). Epidemiological data indicate that while the prevalence of sleep disorders in the general population is approximately 27.0% (Chattu et al., 2018), it is 39.2% in healthcare workers (Qiu et al., 2020) and alarmingly high (59.3–73.3%) in ED nurses (Weaver et al., 2016; Zhang H. et al., 2025). Poor sleep quality in ED nurses impairs cognitive functions such as attention and memory and reduces procedural accuracy and emergency response capabilities, directly impacting patient safety and care quality (García-Tudela et al., 2023). Thus, identifying the factors associated with sleep disorders in ED nurses is crucial for improving care quality and safeguarding their long-term health and professional performance.

Existing research (Weaver et al., 2016; García-Tudela et al., 2023; Zhang W. X. et al., 2025) on nurses’ sleep quality primarily relies on mean scores derived from standardized self-report instruments, with a focus on group-level differences. This variable-centered analytical approach obscures meaningful inter-individual variations in sleep characteristics and fails to identify latent subtypes of sleep quality. This highlights the need to adopt person-centered methodologies—such as latent profile analysis (LPA)—which enable the identification of distinct subgroups based on patterns across multiple sleep-related indicators, offering a more holistic understanding of sleep profiles in nursing populations. In recent years, LPA has been increasingly applied to research on sleep problems in nurses. For instance, Han et al. (2020) identified two latent classes—high- and low-symptomatic sleep disturbance—based on sleep disturbance characteristics in newly hired nurses (≤ 2 years of practice) and reported that the high-symptomatic group had significantly higher turnover rates. Pan et al. (2024) classified psychiatric nurses into good, moderate, and poor sleep-quality groups. Likewise, Wei et al. (2025) conducted an LPA of 1,686 clinical nurses in China and identified high, moderate, and low sleep quality patterns. Together, these studies demonstrate substantial inter-individual heterogeneity in sleep conditions in nursing populations and support the applicability of person-centered analytical approaches in this field.

Existing studies have focused predominantly on newly hired, psychiatric, and general clinical nurses, with few focusing on ED nurses. ED nurses are chronically exposed to unique occupational stressors—including heavy rescue workloads, a fast-paced work environment, irregular rotating shifts, and frequent exposure to traumatic events. Therefore, their sleep problems may exhibit latent patterns distinct from those of other nurses; moreover, the factors associated with different sleep subtypes in this population remain unclear.

Psychological resilience, the capacity to maintain equilibrium and adapt effectively to adversity, is a crucial protective factor for mental and physical health (Vella and Pai, 2019). Substantial evidence links higher resilience with improved sleep quality across diverse populations (Liu et al., 2025; Zhang H. et al., 2025). Individuals with greater resilience manage stress more effectively, which is a key regulator of sleep. This is mediated by adaptive cognitive and emotional processes, including positive reappraisal, problem-focused coping, and effective emotion regulation, which buffer against stress-induced sleep disturbances (Spielman et al., 1987). However, research has predominantly examined linear, bivariate associations, often overlooking potential heterogeneity in sleep quality patterns. Few studies have explored how resilience varies across distinct subgroups defined by different sleep profiles. To address this, this study used LPA to identify meaningful sleep quality subgroups in ED nurses. Subsequently, it investigated how these latent classes were associated with general demographic factors and psychological resilience levels. These findings provide an empirical basis for identifying high-risk individuals within this healthcare population and for developing targeted interventions to improve sleep health in emergency nursing.

## 2 Materials and methods

### 2.1 Setting and participants

ED nurses were recruited using convenience sampling from 12 tertiary hospitals in Jinan, Shandong Province, China, between October and December 2025. Eligible participants were registered nurses with at least 1 year of ED experience who provided informed consent and volunteered to participate. Exclusion criteria included nursing interns, those on leave or away for training during data collection, and individuals with cognitive impairments or psychiatric conditions.

### 2.2 Sample size

The required sample size was determined based on the following formula:

$$N=\frac{{Z^{2}}_{1-\alpha {/}_{2}}P(1-P)}{d^{2}}$$

The critical value of Z _1 − α/2_ for the standard normal distribution was 1.96. A prior study reported a 59.3% prevalence of poor sleep quality among ED nurses (Zhang W. X. et al., 2025). With a margin of error (d) of 0.05, the calculated sample size was 370. After accounting for an anticipated 20% attrition rate, the final minimum required sample size was 464.

### 2.3 Research instruments

#### 2.3.1 Demographic questionnaire

A self-developed questionnaire was used to collect demographic and work-related data. The instrument included sex, age, work experience (years), educational level, marital status, monthly income, and number of monthly night shifts.

#### 2.3.2 Connor–Davidson resilience scale (CD-RISC)

The Connor–Davidson Resilience Scale (CD-RISC), originally developed by Connor and Davidson (2003), was adapted into Chinese by Yu and Zhang (2007). Its 25 items are distributed across three dimensions: strength, tenacity, and optimism. Each item is rated on a 5-point Likert scale from 0 (never) to 4 (always), yielding a total possible score between 0 and 100, where higher scores reflect greater psychological resilience. In this study, the CD-RISC demonstrated excellent internal consistency, with a Cronbach’s α of 0.90.

#### 2.3.3 Pittsburgh sleep quality index (PSQI)

The Pittsburgh Sleep Quality Index (PSQI) was originally developed by Buysse et al. (1989) and translated into Chinese by Liu and Tang (1996). It comprises 19 self-rated items across seven domains of sleep quality: subjective sleep quality, sleep latency, sleep duration, habitual sleep efficiency, sleep disturbances, use of sleeping medications, and daytime dysfunction. Each domain is scored from 0 to 3, yielding a total score of 0–21, with higher scores indicating poorer sleep quality. The optimal cutoff score for the PSQI varies across populations (Pan et al., 2024). In studies involving nurses, a total score of ≤ 5 is commonly used to indicate good sleep quality, whereas a score of > 5 indicates poor sleep quality (Zhou et al., 2023). In the present study, the PSQI demonstrated excellent internal consistency, with a Cronbach’s α of 0.89.

### 2.4 Data collection

The electronic questionnaire was developed on an online survey platform and distributed via WeChat (Tencent Holdings Ltd., Shenzhen, China) to ED nurses in the participating hospitals. A total of 672 questionnaires were distributed, of which 36 duplicate responses with > 80% identical answer patterns and 25 responses with excessively short completion times (< 120 s) were excluded, yielding 611 valid responses for analysis (effective response rate 90.9%).

### 2.5 Statistical analysis

Statistical analyses were performed using SPSS 27.0 and Mplus 8.0. LPA was employed to identify distinct sleep-quality subgroups. To determine the optimal number of latent classes, models with ahigh number of classes were sequentially fitted, beginning with a one-class model, and evaluated using model fit indices and interpretability. The fit indices were Akaike information criterion (AIC), Bayesian information criterion (BIC), and sample-size-adjusted BIC (aBIC), with lower values indicating superior fit. Classification accuracy was assessed using entropy, with values exceeding 0.8 representing high-confidence classification (accuracy > 90%). Furthermore, the likelihood ratio test (LRT) and bootstrapped likelihood ratio test (BLRT) were used to compare k-class and (k − 1)-class models; a significant result (*p* < 0.05) suggests that the k-class model provides a better fit than the (k − 1)-class model (Nylund et al., 2007; Tein et al., 2013). Continuous variables (mean ± standard deviation [SD]) were compared using one-way analysis of variance. Categorical variables (frequencies and percentages) were compared using the χ^2^ test or Fisher’s exact test, as appropriate. Statistically significant variables in these univariate analyses were subsequently entered into the logistic regression model to identify independent factors associated, with latent sleep quality profiles with *p* < 0.05 considered statistically significant.

### 2.6 Ethical considerations

This study was approved by the Institutional Review Board of Shandong Provincial Hospital Affiliated to Shandong First Medical University (Approval No.: 2025–864). All participants provided electronic informed consent after being fully informed about the study’s purpose and procedures. The questionnaire was completed anonymously to ensure confidentiality and protect participant privacy. This study adhered to the ethical standards of the Declaration of Helsinki and followed established guidelines for responsible research and scholarly publication.

## 3 Results

### 3.1 General demographic characteristics

The data from 611 ED nurses were analyzed in this study. They were aged 22–45 years, with a mean age of 33.82 ± 6.95 years. The mean CD-RISC score was 79.20 ± 12.37 and that of the PSQI global score was 7.33 ± 4.58. Subscale scores were as follows: sleep latency (1.28 ± 1.03), sleep duration (1.27 ± 0.90), habitual sleep efficiency (0.92 ± 0.79), sleep disturbances (1.05 ± 0.71), subjective sleep quality (1.19 ± 0.97), use of sleeping medications (0.15 ± 0.36), and daytime dysfunction (1.47 ± 0.97). Other demographic and occupational characteristics are presented in Table 1.

**Table 1:** Univariate analysis of factors associated with latent profiles of sleep quality in emergency department nurses (*n* = 611).

Variables	Total sample (*N* = 611)	C1 *n* = 49 (8.0%)	C2 *n* = 92 (15.1%)	C3 *n* = 163 (26.7%)	C4 *n* = 307 (50.2%)	χ^2^/*F*	*p*
	*n* (%)						
Age (years)							
≤29	195 (31.9)	37	8	51	99	115.26	<0.01**
30–39	202 (33.1)	8	19	77	98		
≥40	214 (35.0)	4	65	35	110		
Sex							
Male	164 (26.8)	9	26	39	90	3.55	0.314
Female	447 (73.2)	40	66	124	217		
Work experience (years)							
<5	196 (32.0)	21	31	51	93	27.66	<0.01**
5–10	232 (38.0)	23	33	78	98		
> 10	183 (30.0)	5	28	34	116		
Educational level							
Junior college	242 (39.6)	42	69	67	64	139.55	<0.01**
Bachelor’s degree	326 (53.4)	6	17	87	216		
Master’s degree	43 (7.0)	1	6	9	27		
Marital status							
Unmarried	209 (34.2)	17	36	53	103	1.26	0.738
Married	402 (65.8)	32	56	110	204		
Monthly income (CNY)							
<5,000	232 (38.0)	33	55	55	89	75.81	<0.01**
5,000–10,000	197 (32.2)	15	34	49	99		
>10,000	182 (29.8)	1	3	59	119		
Monthly night shift number							
0	142 (23.2)	1	12	37	92	98.98	<0.01**
1–4	159 (26.0)	3	17	42	97		
5–8	157 (25.7)	12	23	43	79		
>8	153 (25.1)	33	40	41	39		
Psychological resilience	611 (100.0)	58.33 ± 5.54	64.12 ± 6.10	75.79 ± 8.01	88.87 ± 4.13	720.54	<0.01**

C1, “severely disturbed sleep” profile; C2, “sleep onset difficulty-sleep medication use” profile; C3, “mildly disturbed sleep” profile; C4, “good sleep quality” profile. * *p* < 0.05, ** *p* < 0.01.

### 3.2 Latent profile analysis of sleep quality

LPA of sleep quality was conducted using the seven subscale scores of the PSQI as observed indicators. Five latent profile models were fitted, with the number of latent classes increasing from 1 to 5. As the number of classes increased, the AIC, BIC, and aBIC decreased progressively. When comparing the two-class and the three-class models, both the LRT and BLRT were statistically significant (*p* < 0.05), with entropy > 0.8. Similarly, the comparison between the three- and four-class models showed significant LRT and BLRT values (*p* < 0.05), with entropy > 0.8. However, when moving from the four- to five-class model, the LRT was no longer significant (*p* > 0.05), indicating no meaningful improvement in model fit (Table 2). From a clinical perspective, the four-class model offered greater interpretability than the three-class model, as it identified a distinct profile—“severely disturbed sleep”—a high-risk subgroup that would have been obscured in models with fewer classes. The four-class model was therefore retained as the optimal model based on statistical fit and clinical interpretability.

**Table 2:** Latent profile analysis of sleep quality in emergency department nurses (*n* = 611).

Model	AIC	BIC	aBIC	Entropy	*p*		Proportions
					LRT	BLRT	
1	10054.149	10115.960	10071.513	-	-	-	1.000
2	7184.299	7281.432	7211.586	0.998	0.974	<0.001	0.849/0.151
3	5364.056	5496.509	5401.265	0.991	0.009	<0.001	0.504/0.345/0.151
4	5063.513	5231.287	5110.645	0.981	0.014	<0.001	0.151/0.080/0.498/0.271
5	4708.379	4911.473	4765.433	0.997	0.286	<0.001	0.163/0.195/0.349/0.142/0.151

AIC, Akaike information criterion; BIC, Bayesian information criterion; aBIC, adjust Bayesian information criterion; LRT, likelihood ratio test; BLRT, bootstrapped likelihood ratio test.

The analysis identified four latent classes with distinct sleep-quality profiles (Figure 1). Class 1, labeled “severely disturbed sleep,” displayed the highest scores for sleep duration, sleep disturbances, subjective sleep quality, and daytime dysfunction, indicating profoundly impaired sleep. Characterized by the longest sleep latency and greatest reliance on sleep medications, Class 2 was designated the “sleep onset difficulty-sleep medication use” profile. Class 3 showed moderately elevated scores across most PSQI dimensions, exceeding the sample mean without reaching severe impairment, and was labeled the “mildly disturbed sleep” profile. In contrast, Class 4 exhibited the lowest scores on all PSQI dimensions, reflecting consistently good sleep quality, and was classified as the “good sleep quality” profile.

**Figure 1:**
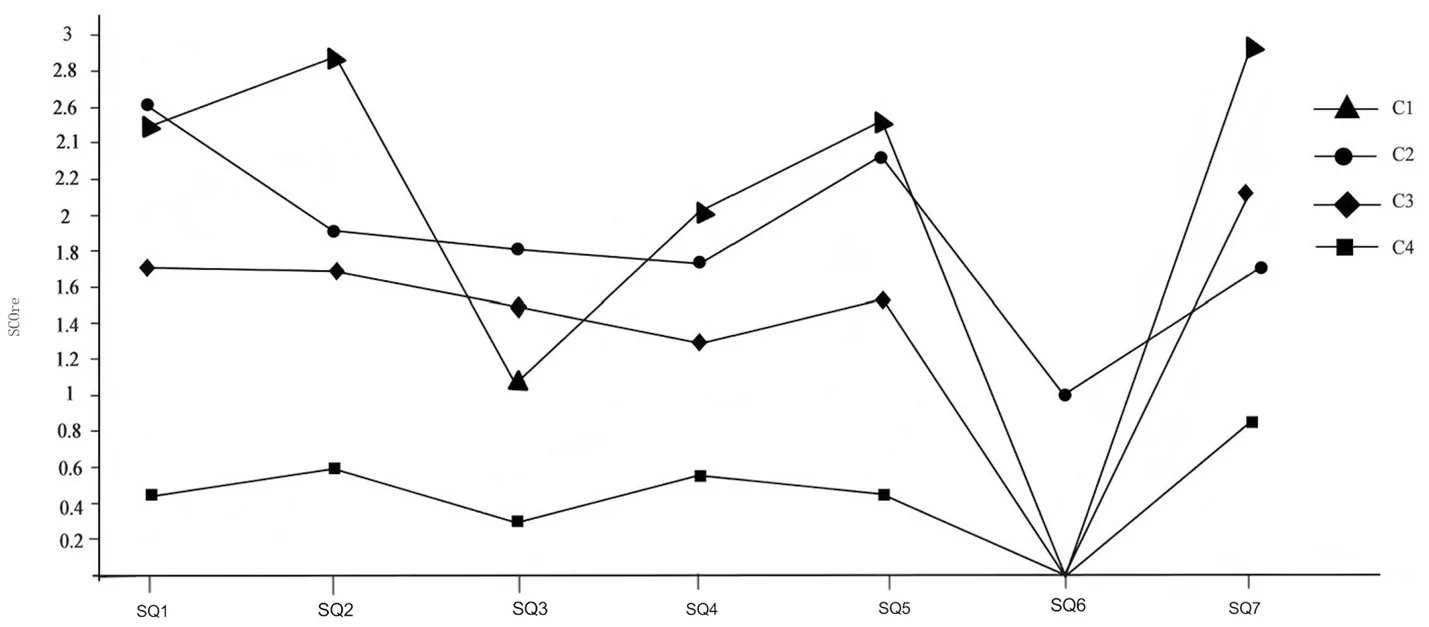
Four latent profile classes of sleep quality in emergency department nurses. SQ1, sleep latency; SQ2, sleep duration; SQ3, habitual sleep efficiency; SQ4, sleep disturbances; SQ5, subjective sleep quality; SQ6, use of sleeping medications; SQ7, daytime dysfunction; C1, "severely disturbed sleep" profile; C2, "sleep onset difficulty-sleep medication use" profile; C3, "mildly disturbed sleep" profile; C4, "good sleep quality" profile.

### 3.3 Univariate analysis of factors associated with sleep quality profiles

A univariate analysis was conducted using the four latent profiles as the dependent variable. The results revealed significant differences across profile groups in age, work experience, educational level, monthly income, monthly night shift number, and psychological resilience (*p* < 0.01) (Table 1).

### 3.4 Multivariate logistic regression analysis of factors associated with sleep quality profiles

A multinomial logistic regression analysis was performed using variables that were significant in the univariate analyses and Class 4 as the reference. As shown in Table 3, psychological resilience, age, work experience, monthly income, and monthly night shift number were significantly associated with latent class membership.

**Table 3:** Logistic regression analysis of latent profiles of sleep quality in emergency department nurses (*n* = 611).

Variables	C1 vs. C4					C2 vs. C4					C3 vs. C4				
	B	SE	*p*	OR	95% CI	B	SE	*p*	OR	95% CI	B	SE	*p*	OR	95% CI
Psychological resilience	**−0.76**	**0.07**	**<0.001**	**0.47**	**(0.41, 0.54)**	**−0.61**	**0.05**	**<0.001**	**0.54**	**(0.49, 0.60)**	**−0.38**	**0.04**	**<0.001**	**0.68**	**(0.64, 0.74)**
Age (years) (ref: ≥40)															
≤29	**0.12**	**0.95**	**0.025**	**8.35**	**(1.30, 53.67)**	**−2.02**	**0.76**	**0.007**	**0.13**	**(0.03, 0.58)**	0.71	0.44	0.102	2.04	(0.87, 4.81)
30–39	−0.23	0.94	0.805	0.79	(0.13, 5.03)	**−1.63**	**0.66**	**0.013**	**0.20**	**(0.05, 0.71)**	**0.90**	**0.42**	**0.033**	**2.45**	**(1.08, 5.57)**
Work experience (years) (ref: >10)															
<5	**2.65**	**1.03**	**0.010**	**14.12**	**(1.88, 106.36)**	1.11	0.71	0.120	3.03	(0.75, 12.27)	0.91	0.47	0.055	2.48	(0.98, 6.27)
5–10	**3.22**	**1.05**	**0.002**	**25.13**	**(3.20, 197.15)**	1.27	0.70	0.070	3.56	(0.90, 14.12)	**1.06**	**0.43**	**0.015**	**2.89**	**(1.23, 6.75)**
Educational level (ref: Master’s degree)															
Junior college	0.72	1.58	0.648	2.05	(0.09, 44.97)	0.98	1.09	0.365	2.68	(0.32, 22.49)	1.21	0.65	0.063	3.35	(0.94, 11.93)
Bachelor’s degree	−0.52	1.63	0.749	0.59	(0.03, 14.37)	−0.90	1.11	0.417	0.41	(0.05, 3.56)	0.31	0.64	0.624	1.37	(0.39, 4.74)
Monthly income (CNY) (ref: >10,000)															
<5,000	**3.64**	**1.41**	**0.010**	**38.11**	**(2.38, 609.28)**	**2.67**	0.**87**	0.**002**	**14.41**	**(2.65, 78.47)**	0.26	0.40	0.519	1.29	(0.59, 2.82)
5,000–10,000	**3.38**	**1.45**	**0.020**	**29.45**	**(1.72, 504.72)**	**2.49**	0.**89**	0.**005**	**12.11**	**(2.12, 69.21)**	0.18	0.41	0.665	1.19	(0.54, 2.67)
Monthly night shift number (ref: >8)															
0	**−4.25**	**1.28**	**0.001**	0.**01**	**(0.01, 0.18)**	**−2.79**	**0.80**	**0.001**	**0.06**	**(0.01, 0.30)**	**−1.34**	**0.50**	**0.007**	**0.26**	**(0.10, 0.70)**
1–4	**−3.39**	**1.12**	**0.002**	0.**03**	**(0.01, 0.30)**	**−1.87**	**0.75**	**0.013**	**0.15**	**(0.04, 0.67)**	−0.78	0.50	0.116	0.46	(0.17, 1.21)
5–8	−2.83	0.93	0.002	0.06	(0.01, 0.36)	**−2.01**	**0.74**	**0.007**	**0.13**	**(0.03, 0.57)**	−0.69	0.48	0.154	0.50	(0.19, 1.29)

All predictor variables are highlighted in bold. C1, “severely disturbed sleep” profile; C2, “sleep onset difficulty-sleep medication use” profile; C3, “mildly disturbed sleep” profile; C4, “good sleep quality” profile.

Higher psychological resilience was associated with significantly lower odds of being assigned to any of the three sleep disturbance profiles (odds ratio [OR] = 0.47, 95% confidence interval [CI]: 0.41–0.54; OR = 0.54, 95% CI: 0.49–0.60; OR = 0.68, 95% CI: 0.64–0.74). The strongest inverse association was observed for the “severely disturbed sleep” profile, corresponding to 53.2% lower odds of classification into this group. Participants aged 29 years or younger were significantly more likely to be classified into Class 1 (OR = 8.35, 95% CI: 1.30–53.67). In contrast, both younger age groups showed markedly lower odds of belonging to Class 2: compared to those aged 40 years or older, those aged ≤29 years had 86.8% lower odds (OR = 0.13, 95% CI: 0.03–0.58) and those aged 30–39 years had 80.5% lower odds (OR = 0.20, 95% CI: 0.05–0.71). Conversely, nurses aged 40 years or older were most likely to be assigned to Class 2. Additionally, nurses aged 30–39 years were significantly more likely than their older counterparts to be classified into Class 3 (OR = 2.45, 95% CI: 1.08–5.57).

Compared to nurses with more than 10 years of work experience, those with shorter tenure were significantly more likely to be classified into Class 1. Specifically, nurses with 5–10 years of experience had 14.1 times the odds (OR = 14.12, 95% CI: 1.88–106.36) and those with ≤ 5 years, 25.1 times the odds (OR = 25.13, 95% CI: 3.20–197.15) of belonging to this profile. Additionally, nurses with 5–10 years of experience showed higher odds of being assigned to Class 3 (OR = 2.89, 95% CI: 1.23–6.75).

Nurses with monthly incomes below 10,000 CNY were significantly more likely than those with higher income to be assigned to either Class 1 (OR = 38.11, 95% CI: 2.38–609.28; OR = 29.45, 95% CI: 1.72–504.72) or Class 2 (OR = 14.41, 95% CI: 2.65–78.47; OR = 12.11, 95% CI: 2.12–69.21). Compared to nurses working more than eight night shifts per month, those working ≤ 4 night shifts per month were significantly less likely to be classified into Class 1 (OR = 0.01, 95% CI: 0.01–0.18; OR = 0.03, 95% CI: 0.01–0.30). Having ≤ 8 night shifts per month was significantly inversely associated with classification into Class 2 (OR = 0.06, 95% CI: 0.01–0.36; OR = 0.15, 95% CI: 0.04–0.67; OR = 0.13, 95% CI: 0.03–0.57). Furthermore, not having any night shifts was significantly associated with lower odds of being assigned to Class 3 (OR = 0.26, 95% CI: 0.10–0.70).

## 4 Discussion

In the present study, ED nurses reported a mean sleep quality score of 7.33 ± 4.58, indicating generally poor sleep quality. This finding is consistent with previous studies (Qiu et al., 2020; Aljohani et al., 2021; O’Bryon et al., 2025), underscoring that suboptimal sleep is a prevalent issue among ED nurses both domestically and internationally. This may be attributed to the demanding nature of the work, including shift work, severity of patients’ conditions, and irregular scheduling and intrinsic stressors associated with the professional role (García-Tudela et al., 2023; Bekircan et al., 2025).

Furthermore, this study revealed four distinct sleep-quality profiles among ED nurses: (1) severely disturbed sleep, (2) sleep onset difficulty-sleep medication use, (3) mildly disturbed sleep, and (4) good sleep quality. These heterogeneous profiles suggest that ED nurses do not exhibit a uniform pattern of sleep quality. Recognizing this heterogeneity is crucial for developing targeted interventions tailored to each subgroup rather than adopting a one-size-fits-all approach to sleep health.

In the present study, the “severely disturbed sleep” profile was more likely to comprise nurses aged 29 years or younger with shorter work experience (particularly ≤ 5 years), monthly income below 10,000 CNY, and more than eight night shifts per month. These findings suggest an overrepresentation of early-career nurses. This association may be partly attributable to the relatively immature circadian regulation and less developed stress-coping mechanisms, which may be compounded by frequent night shifts and financial pressure (Liu et al., 2025; Zhang H. et al., 2025); however, given the cross-sectional design, these interpretations remain speculative and warrant verification in future longitudinal studies. Both higher psychological resilience and ≤ 4 night shifts per month were significantly associated with lower odds of belonging to this high-risk profile, suggesting that these factors may serve as potential protective correlates. Previous research indicates that psychological resilience, a key resource for coping with stress and adversity, may provide a critical buffer in high-demand occupational settings such as night shifts (Dong et al., 2025). Higher resilience may enable healthcare professionals to regulate their emotions more effectively and maintain cognitive function, thereby sustaining relatively stable psychological states despite challenges such as circadian disruption, role conflict, and physical fatigue (Şimşek et al., 2025). Such stability has been linked to a lower risk of mental health issues, including anxiety, depression, and burnout (Vella and Pai, 2019). Similarly, limiting night shifts to four per month may help preserve circadian rhythms, potentially mitigate the cognitive impairments and metabolic disturbances associated with sleep deprivation, and allow sufficient recovery time that may contribute to a healthier work-life balance (Yang et al., 2025). Based on these associations, potential measures include restricting night shift frequency during initial employment, incorporating psychological resilience training such as mindfulness-based programs, providing financial support, and establishing routine sleep health screenings, all of which may facilitate occupational adaptation and long-term well-being.

The “sleep onset difficulty-sleep medication use” profile primarily comprised nurses aged 40 years and older with longer work tenures. In contrast, younger nurses—particularly those aged 29 or below—were significantly less likely to belong to this category, suggesting that sleep problems accompanied by medication use may be more frequently observed with advancing age. This finding is consistent with previous research (de Bruin et al., 2017) which suggests that age-related alterations in sleep architecture, such as reduced slow-wave sleep, increased nocturnal awakenings, and weakened circadian rhythm regulation, may contribute to this pattern. This profile was also more prevalent in nurses earning less than 10,000 CNY per month, suggesting that financial stress may be associated with a greater reliance on sleep medications. A frequent night-shift schedule (more than eight per month) was significantly associated with this profile and may be regarded as a potential risk correlate. Conversely, a lower night-shift frequency was associated with lower odds of belonging to this profile, suggesting a potential protective association; accordingly, irregular or high-intensity shift patterns may be associated with greater use of sleep aids, possibly owing to circadian disruption and increased psychological strain (García-Tudela et al., 2023). Notably, psychological resilience was associated with lower odds of belonging to all three sleep disturbance profiles; however, its association appeared weaker in this subtype. This suggests that once a pattern of regular sleep medication use is established, enhancing psychological adaptability alone may be insufficient to reverse this behavioral pattern (Wang et al., 2021). Targeted strategies for senior nurses should therefore consider emphasizing non-pharmacological sleep interventions, rational shift scheduling, and economic support, which may help reduce the risk of continued reliance on sleep medications.

The “mildly disturbed sleep” profile was more likely to comprise nurses aged 30–39 years with 5–10 years of work experience, who may face concurrent career and family pressures. Although their sleep issues were not severe, early signs of sleep disturbance may already be apparent. Complete avoidance of night shifts was significantly associated with lower odds of classification into this subtype, suggesting that minimizing night-shift exposure may be protective. This association may be partly attributable to the possibility that regular sleep patterns help stabilize circadian rhythms and minimize cortisol fluctuations (Rosa et al., 2019). Furthermore, psychological resilience was consistently associated with lower odds of having all sleep disturbance profiles, suggesting a potential protective association across subtypes. Thus, early interventions should be considered for this at-risk group, including psychological resilience training, flexible shift scheduling, and integrating sleep health into routine occupational health management. These measures may help prevent mild sleep disturbances from progressing and shift care from treatment toward prevention.

The allostatic load theory provides a biological account of the protective role of psychological resilience observed across the profiles (McEwen and Karatsoreos, 2022). Psychological resilience may buffer the translation of occupational stress into physiological burden: individuals with higher resilience tend to perceive stressors as more controllable and adopt adaptive coping strategies, thereby attenuating hypothalamic–pituitary–adrenal axis reactivity, preserving cortisol rhythmicity and melatonin secretion, and consequently, mitigating the circadian disruption and neuroinflammation that destabilize sleep homeostasis (McEwen and Karatsoreos, 2022). Psychological resilience is also associated with lower pre-sleep cognitive arousal and rumination—key processes in the hyperarousal model of insomnia—and with behaviors facilitating sleep onset and reduce arousal, limiting the accumulation of allostatic load (McEwen and Karatsoreos, 2022; Ali et al., 2025). This multi-level buffering effect may explain why higher psychological resilience was consistently associated with lower odds of belonging to any sleep disturbance profile. Notably, the strength of this association varied across profiles and was weaker in the “sleep onset difficulty-sleep medication use” profile. This pattern suggests that psychological resources alone may be insufficient to address established maladaptive behaviors, such as medication dependence, or accumulated allostatic load. Rather, the protective association of psychological resilience may be primarily observed at the early stages of the stress–sleep pathway, and may be inversely associated with more severe sleep disturbances or states of regular sleep medication use (Irwin et al., 2016; Kent de Grey et al., 2018). These findings suggest that resilience-building programs may be beneficial for nurses across all profiles, potentially by reducing the accumulation of allostatic load.

### 4.1 Limitations

This study identified latent profiles of sleep quality among ED nurses and their associated factors; however, it has several limitations. First, participants were recruited from tertiary hospitals in Shandong Province, China, using convenience sampling, which may limit the representativeness of the sample and constrain the generalizability of the findings to other regions or healthcare settings of different levels. However, Shandong Province, as one of the most populous provinces in China, has demographic structures, economic development levels, and healthcare resource allocation that are broadly representative of the national context; therefore, the findings may serve as a reference for similar regions and tertiary hospitals. Second, the proportion of participants classified into the “severely disturbed sleep” profile was small (approximately 8%), which may have resulted in unstable inferential estimates for this subgroup. Future studies with larger sample sizes are warranted to further explore the associated factors and clinical implications of severe sleep disturbance. Third, the cross-sectional design allowed for the detection of associations among variables but precluded causal inference and longitudinal effects of these factors on distinct sleep quality profiles. Finally, the data were collected through self-administered questionnaires; therefore, the findings may be susceptible to recall bias and common method bias. Future studies should employ objective sleep assessment instruments and multi-source data collection approaches and adopt longitudinal designs to further elucidate causal relationships among the variables.

### 4.2 Implications for emergency nurses

This cross-sectional study identified four heterogeneous sleep quality profiles in ED nurses, suggesting that a uniform intervention strategy may be insufficient and that tailored, precision-oriented management approaches could be more appropriate. The findings suggest that young nurses, who exhibited a higher prevalence of the “severely disturbed sleep” profile potentially linked to frequent night shifts, lower income, and poorer psychological adjustment, may benefit from early protective strategies such as optimizing night shift frequency, providing psychological resilience training, and improving economic support. Middle-aged nurses may face concurrent professional and familial demands; they predominantly presented with the “mildly disturbed sleep” profile yet represent a potentially high-risk subgroup; flexible scheduling and preventive psychological interventions may be warranted to prevent further deterioration. For nurses with the “sleep onset difficulty-sleep medication use” profile, enhancing psychological resilience alone may be inadequate, and non-pharmacological approaches could be promoted alongside optimized shift schedules to reduce reliance on hypnotics. Furthermore, the results highlight the potential adverse associations between occupational factors (e.g., frequent night shifts and lower income) and sleep quality. This underscores the need for nursing managers to consider implementing a rational shift system that limits consecutive night shifts and ensures adequate rest periods and improving the salary structure to enhance job satisfaction.

## 5 Conclusion

This study found poor overall sleep quality among ED nurses. LPA revealed four distinct latent classes of sleep quality: (1) severely disturbed sleep, (2) sleep onset difficulty-sleep medication use, (3) mildly disturbed sleep, and (4) good sleep quality. This classification indicates significant heterogeneity in sleep quality within this group. Psychological resilience, age, work experience, monthly income, and monthly night shift number were key factors associated with the classification of emergency nurses’ sleep quality categories.

## Data Availability

The raw data supporting the conclusions of this article will be made available by the authors, without undue reservation.
